# Orthodontics and veneers to restore the anterior guidance. A minimally invasive approach

**DOI:** 10.4317/jced.54358

**Published:** 2017-11-01

**Authors:** Vicente Faus-Matoses, Ignacio Faus-Matoses, Ana Jorques-Zafrilla, Vicente J. Faus-Llácer

**Affiliations:** 1 DDS, MSc, PhD. Co-director of the Master of Restorative Dentistry and Endodontics, Department of Stomatology, Medicine and Dental School, Valencia University, Spain; 2 DDS, MSc, PhD. Professor of the Master in Orthodontics, Department of Stomatology, Medicine and Dental School, Valencia University, Spain; 3 Postgraduate Student, Valencia University, Spain; 4 MD, DDS, PhD. Director of the Master of Restorative Dentistry and Endodontics, Department of Stomatology, Medicine and Dental School, Valencia University, Spain

## Abstract

Tooth wear is defined as the progressive loss of a tooth’s surface due to actions other than those which cause tooth decay or dental trauma. It is a pathological condition with an increasing prevalence among young people. The aim of this article is to describe an alternative treatment modality to rehabilitate the anterior guidance by a minimally invasive interdisciplinary ortho-restorative treatment. Two patients came to the dental clinic for restorative treatment in order to rehabilitate the worn anterior dentition. Clinical analysis showed tooth surface loss located at the incisal edges by attrition due to an inadequate anterior guidance. In both cases the occlusal vertical dimension was reduced. First, following Dahl’s principle, resin attachments were placed in the upper canines. These attachments allowed the extrusion of posterior teeth in order to increase the occlusal vertical dimension. Furthermore, anterior teeth were intruded in order to create space for the restorative material. In the second phase, the restorative treatment was completed. Due to the characteristics of the case, feldspathic ceramic veneers were indicated. A diagnostic wax-up was performed to assist the treatment planning and a mock-up was prepared. Then, maxillary incisors were prepared through the mock-up to ensure a minimally invasive technique. Afterwards, silicone impressions were taken. Finally, veneers were cemented with a light-cured cement. In the present case, the functional and aesthetic parameters required by the patients were achieved, thus satisfying their needs.

** Key words:**Tooth wear, anterior guidance, feldspathic veneers, Dahl’s principle, minimally invasive.

## Introduction

Tooth wear is defined as the loss of tooth substance in the absence of caries and plaque. It is a normal physiological process and occurs throughout life but is considered pathological when the degree of destruction is excessive or the rate of loss is rapid, causing functional, aesthetic or sensitivity problems (1). Tooth surface loss can present in various clinical forms with a wide range of etiological factors (2).

Occlusal wear of teeth due to attrition is the result of friction by functional and parafunctional activities. Occlusal, incisal and interproximal surfaces can be affected by attrition (1,3). Frictional tooth wear alters the existing occlusal plane introducing occlusal interferences (3).

Traditionally, a full-mouth rehabilitation based on full-crown coverage has been the recommended treatment for patients affected by dental wear (4,5). However, in recent years, there has been an increasing trend towards the restoration of worn dentitions using more conservative approaches with adhesive restorative materials, such as direct composite restorations or veneers (3-5). These approaches aim to preserve as much remaining tooth structure as possible (2-6).

The restoration of the anterior teeth using ceramic veneers of minimum thickness is a minimally invasive treatment option. It has been used due to ceramics’ color stability, biocompatibility, mechanical properties, and aesthetic outcome (6).

The aim of this article is to describe an alternative treatment modality to rehabilitate the anterior guidance by a minimally invasive interdisciplinary ortho-restorative treatment.

## Case Reports

CASE 1

**Figure 1:**
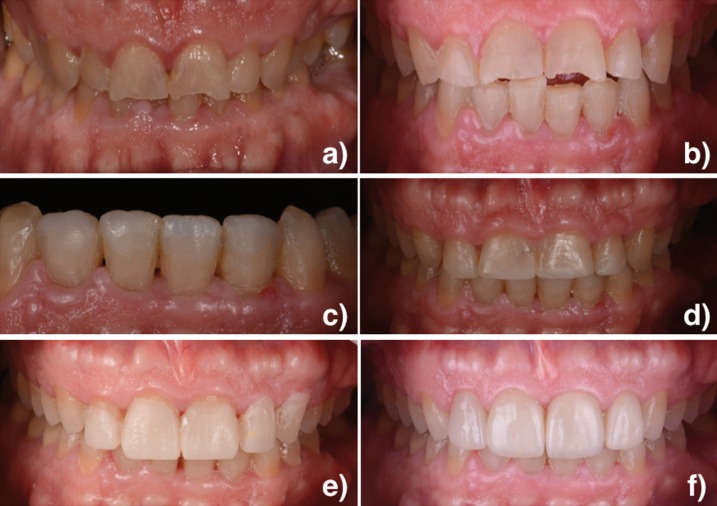
a) Initial situation. b) After orthodontic treatment situation. c) Direct composite restorations from 32 to 42. d) Direct composite restorations from 12 to 22. e) Mock-up. f) Final situation.

A 43-year-old male patient was referred to the dental clinic. Clinically, tooth wear was only diagnosed in the anterior region due to an inadequate anterior guidance, thus a consequent reduction of the occlusal vertical dimension (Fig. 1a).

CASE 2

**Figure 2:**
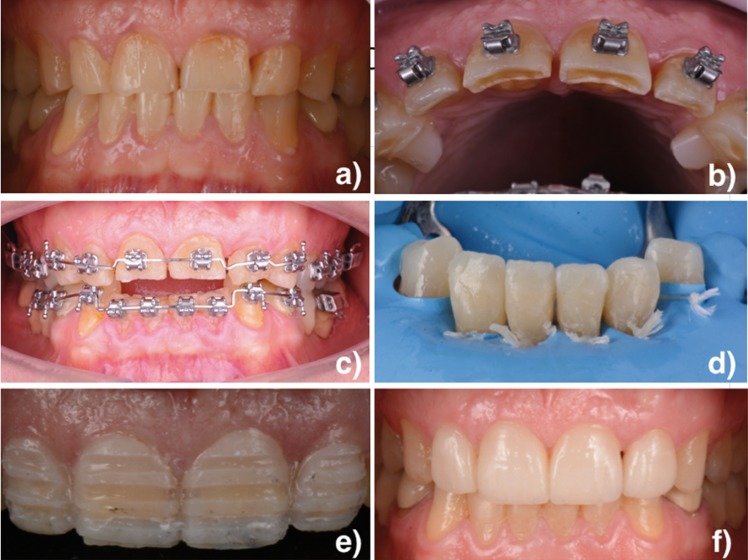
a) Initial situation. b) Resin attachments. c) Orthodontic treatment. d) Direct composite restorations from 32 to 42. e) Preparation through the mock-up. f) Final situation.

A 42-year-old male patient came to the dental clinic. He complained of discomfort caused by his worn anterior teeth. Clinical analysis showed tooth wear with dentine exposure and a reduction in clinical crown height. He presented an incisal edge-to-edge relationship (Fig. 2a).

In both cases, anterior teeth were worn and the oclussal vertical dimension was reduced. After clinical examination, impressions of maxillary and mandible arches were taken with alginate to obtain preliminary casts. After thorough diagnosis and planning, a comprehensive treatment plan that incorporated all the wishes of the patient was devised. In both cases an interdisciplinary ortho-restorative treatment was carried out in order to ensure a minimally invasive treatment.

In the first phase, orthodontic treatment was carried out. Following Dahl’s principle, resin attachments (Mini-Mold bite opening kit, G&H Orthodontics, Franklin, Indiana, EEUU) were placed in the upper canines (Fig. 2b). These attachments allowed the extrusion of posterior teeth in order to increase occlusal vertical dimension. At the same time, anterior teeth were intruded in order to create space for the restorative material (Fig. 1b, Fig. 2c).

 In the second phase, the restorative treatment was completed. Previously, a diagnostic wax-up was performed to define shape and form to assist the treatment planning. Mandibular incisors were restored by direct composite restorations with Ceram X (Dentsply Sirona, Konstanz, Germany) (Fig. 1c,Fig. 2d). Then, incisal lengthening with direct composite (Ceram X, Dentsply Sirona, Konstanz, Germany) was performed (Fig. 1d). Due to the case characteristics, feldspathic ceramic veneers (Kuraray Noritake dental Inc, Japan) of minimum thickness were indicated for the four maxillary incisors. A mock-up was prepared for the maxillary incisors (Fig. 1e). Its functionality and aesthetics were verified statically and dynamically. By using the mock-up technique, minimal preparations were made to ensure a minimally invasive technique (Fig. 2e). Afterwards, silicone impressions (Aquasil Ultra, Dentsply Sirona, Konstanz, Germany) were taken.

In the following visit, a try-in paste was used to select the proper color of the luting cement. Veneers were then washed to remove try-in paste and carefully air-dried. The veneers’ internal surface was etched with 9% hydrofluoric acid (Ultradent, Ultradent Products. Inc.) for 60 seconds, washed under running water and dried with an air syringe. Then, silane (Calibra, Dentsply Sirona, Konstanz, Germany) and one coat of Prime&Bond NT (Dentsply Sirona, Konstanz, Germany) were applied. At the same time, tooth surface was acid etched (DeTrey Conditioner 36, Dentsply Sirona, Konstanz, Germany) and bonded with Prime&Bond NT (Dentsply Sirona, Konstanz, Germany). Veneers were cemented with a light-cured cement (Calibra Veneer, Dentsply Sirona, Konstanz, Germany). The cement was applied to the veneers that were gently seated with finger pressure. Excess cement was removed. The light polymerization was performed for 40 seconds from buccal, incisal, mesial and distal aspects of each tooth.

The final restorative phase was achieved by checking the restorations for any occlusal interference. Immediate final restorations can be observed in Figures 1f and 2f.

## Discussion

Patients with a worn anterior dentition suffer from a loss of clinical crown height and the possibility of development of an edge-to-edge incisal relationship. As a result, the aesthetic appearance is affected and the anterior guidance is lost (7). In addition, as a result of vertical height loss, the interocclusal space for our restorations may be reduced. In these cases, the best option is to increase the vertical occlusal dimension. It will rectify the anterior tooth relationship, improving aesthetics and facilitating the establishment of anterior tooth guidance (5,8).

Dhal proposed creating space in the treatment of localized anterior tooth wear by separating the posterior teeth through an anterior bite plan for about 4 to 6 months. A combination of passive eruption (posterior teeth) and intrusion (anterior teeth) allowed the reestablishment of posterior occlusion while maintaining the anterior space (2). It has also been suggested restoring the anatomical form of the anterior teeth and leaving the posterior teeth discluded and the subsequent eruption of the unaffected posterior teeth enables equilibration of the occlusion without any further restorative work (4). In both anterior cases, resin attachments were placed in upper canines. These attachments allowed the extrusion of posterior teeth in order to increase occlusal vertical dimension. At the same time, anterior teeth were intruded in order to create space for the restorative material.

Once the vertical dimension has been increased, a favourable incisal guide which complies with the aesthetic and phonetic demands must be restored (9). In recent years, there has been an increasing trend towards the restoration of severely worn dentitions using more conservative approaches with adhesive restorative materials (2-6).

One option is to restore the dentition by direct composite restorations. The use of silicone putty keys for the reconstruction of lost tooth structure is well established. These techniques enable the reconstruction of the palatal and incisal surfaces of the teeth to match the diagnostic wax-up and thereby reduce the need for major occlusal adjustments (4). In the present case, direct composite restorations were performed in order to reestablish the shape and form of the mandibular incisors.

An alternative option is the placement of ceramic veneers. Its thickness and the color of the underlying dental structure must be taken into account. In the present case, the aim was to improve the aesthetics by modifying the shape and length of the clinical crown of the anterior teeth. In these cases, ceramic laminate veneers may be indicated (6).

In conclusion, the ortho-restorative interdisciplinary treatment is the best option in cases of anterior guidance rehabilitation in which minimal invasiveness is required. In the present case, the functional and aesthetic parameters required by the patients were achieved, thus satisfying their needs.
